# Diagnostic Performance and Clinical Applications of a Commercial Alpha‐Synuclein Seed Amplification Assay in a Tertiary Care Memory Clinic

**DOI:** 10.1002/alz.089116

**Published:** 2025-01-09

**Authors:** Amanda Porter, Christian Lachner, Philip W Tipton, Neill R Graff‐Radford, Gregory S Day

**Affiliations:** ^1^ Mayo Clinic, Jacksonville, FL USA; ^2^ Department of Neurology, Mayo Clinic, Jacksonville, FL USA

## Abstract

**Background:**

SAAs represent a promising biomarker of Lewy Body disease (LBD), with high sensitivity (87.3%, 95%CI: 0.755‐0.947) and specificity (97.2%, 95%CI: 0.922‐0.994) for detection of patients in the Parkinson’s Progression Markers Initiative repository with positive DaT SPECT; and reasonable sensitivity (78.7%, 95%CI: 0.66‐0.88) and specificity (89.5%, 95%CI: 0.78‐0.96 referencing clinical diagnoses in participants included within national research repositories. However, test performance in heterogeneous clinical cohorts is unknown.

**Method:**

Patients with typical (n=122) and rapid progressive dementia (n=76) consented to banking of CSF at the time of clinically‐indicated diagnostic lumbar puncture. Clinical data were deidentified and uploaded to a secure research database and clinical (etiologic) diagnoses established by two neurologists following independent review of available records. SAA (SYNTap™) were run on stored CSF by Amprion Diagnostics (San Diego, CA).

**Result:**

Median participant age was 69.1 years (18.4‐86.1); 56.3% were female. CSF was sampled median 1.4 years (0‐10.3) after symptom onset. Pathological alpha‐synuclein was detected in 28/122 patients with typically progressive dementia (including 6/18 patients with presumed LBD; sensitivity 33%, specificity 79%) and 11/76 patients with rapid progressive dementia (including 3/9 patients with presumed LBD; sensitivity 33%, specificity 88%). “Positive” SAA was associated with tremor (OR=3.2, 95%CI: 1.3‐7.8), visual hallucinations (OR=6.14, 95%CI 1.6‐23.6), and REM behavior disorder (OR=3.7, 95%CI: 1.4‐9.5) in patients with typical but not rapid progressive dementia. Brain autopsies were completed in 17 patients: Lewy bodies were detected in 2/3 patients with positive SAA and no patients with negative SAA.

**Conclusion:**

“Positive” SAA demonstrated reasonable specificity but low sensitivity for the diagnosis of LBD. Additional studies in heterogeneous cohorts with neuropathologic data are needed to optimize clinical applications of SAA.

